# Challenges of a Circular Economy: The Example of Raw Recycled Tyre Steel Fibres Added to Concrete

**DOI:** 10.3390/ma17184554

**Published:** 2024-09-17

**Authors:** Agnieszka Michalik, Filip Chyliński, Jacek Zychowicz, Waldemar Pichór

**Affiliations:** 1 Building Structures, Geotechnics and Concrete Department, Building Research Institute, ul. Filtrowa 1, 00-611 Warsaw, Poland; f.chylinski@itb.pl; 2 Faculty of Civil Engineering and Geodesy, Military University of Technology, ul. gen. Sylwestra Kaliskiego 2, 00-908 Warsaw, Poland; jacek.zychowicz@wat.edu.pl; 3 Department of Building Materials Technology, Faculty of Materials Science and Ceramics, AGH University of Science and Technology, al. Mickiewicza 30, 30-059 Cracow, Poland; pichor@agh.edu.pl

**Keywords:** recycled tyre steel fibres (RTSF), manufactured steel fibres (MSF), circular economy, sustainable development, raw tyre fibres, treated tyre fibres

## Abstract

This research was conducted to analyse the possibility of using raw, untreated recycled tyre fibres as an effective concrete reinforcement according to circular economy principles. The aim of the article was also to develop a method for dispensing tire fibres on a real scale. Additional treatment and homogenisation of recycled steel fibres entail higher energy consumption, emissions of greenhouse gases, and increased costs. However, obtaining durable and safe concrete effectively reinforced with steel fibres is critical. Finding a balance between environmental friendliness and product durability is a circular economic challenge. Reference concrete with commercial steel fibres (15 kg/m^3^) and two concretes containing various quantities of non-treated, raw tyre recycled fibres (25 kg/m^3^ and 45 kg/m^3^) were industrially produced. Tests were carried out on the properties of the concrete mixture and hardened concrete, such as compressive strength, flexural strength, splitting strength, modulus of elasticity, residual flexural tensile strength, and fibre distribution in concrete. Tests revealed that increasing the amount of raw tyre fibres disturbs the structure and causes air entrainment and the formation of fibre clusters. Smaller quantities of raw tyre fibres turn out an effective concrete reinforcement. The use of non-treated tyre fibres as concrete reinforcement is possible but requires more stringent control of the concrete parameters. Implementation tests on an industrial scale are a novelty in this study, presenting an analysis of the possible dispensing of tyre fibres in a ready-mixed concrete production plant and testing the characteristics of manufactured concrete.

## 1. Introduction

The problems related to the circular economy using the example of raw recycled tyre steel fibres are presented in this article. Using cleaned and processed tyre fibres matches the measures implemented for sustainable building [1] aimed at using waste materials, saving natural resources, and reducing the carbon footprint by substituting the high-emission production of steel with recycled fibres. Nevertheless, the additional technology involving treating used tyre fibres for rubber and textile contaminants and steel dust and making their dimensions uniform will contribute to higher costs and energy consumption, including a higher carbon footprint.

Literature sources indicate that the cost of recycled tire fibres is approximately 30–70% lower compared to industrial fibres [2,3,4,5]. In this study, the cost of raw RTSF fibres was 38% lower than the cost of MSF fibres.

Therefore, a compromise should be made between treating the fibres and their impact on the strength characteristics of the concrete. The paper presents the need for additional treatment and refining of primarily recycled tyre fibres to be used as dispersed concrete reinforcement according to circular economy principles [3]. The new perspective described in this paper analyses the potential dispensing of tyre fibres on an industrial scale in a ready-mixed concrete plant.

Currently, industrial entities are obliged to take specific measures to counteract hazards caused by environmental and climate crises [4,5]. Moreover, we face the hazard of the scarcity of natural resources. Hence, emphasis is placed on saving natural resources and introducing as much waste and recycled material as possible. The European Union has developed new strategies for sustainable development called the European Green Deal [6], and Fit for 55 [7] has aimed to transform the European Union into a climate-neutral area by 2050, where economic development should be separated from the use of natural resources. It is estimated that only 12% [6] of materials used in the EU’s industrial sector are recycled. The new action plan should support industrial development, for example, with the highest use of waste. Among the assumptions presented in Section 2.1.3, mobilising industry for a clean and circular economy [6] is relevant for the building material sector. The new action plan should support the development of a closed life-cycle and climate-neutral industry, meaning the highest possible industrial use and reuse of waste. Following the above principles, a circular economy model [3] was developed based on three key rules: reduce, reuse, and recycle. The lifecycle of products in a circular economy has been extended. At the end of a product’s life, its raw materials and waste should remain in the economy owing to recycling. In practice, this indicates the maximisation of waste reduction.

One of the most energy-intensive industries is the steel industry, including the production of steel fibres [4]. Therefore, solutions are being sought to reduce the production of new steel. On the other hand, the scale of car tyre production is growing [8] During material recycling, the components of the tire are separated, i.e., rubber, steel, and textiles, which can be reused [9]. The use of secondary resources from tyre material recycling in a circular economy has been the subject of many research projects over the years. Rubber waste [10] has been found to have the broadest applications. It covers several fractions depending on the particle size, that is, cut pieces, shreds, chips, granulate, fines, and pulp. They are used in various sectors, including buildings, road construction, sports and leisure infrastructure, the rubber material industry, and printing [8,10,11,12,13,14], for road pavements (asphalt), railway sleepers, reinforced concrete production (rubber aggregate), lightweight filling in building structures, embankments, road floor layers, noise barrier walls and signs, artificial turf for football pitches, playgrounds, basketball courts, and rubber objects such as mats, doormats, carpets, and support pads for terraces.

Further recycled textile waste is then used in the textile industry, and non-recyclable waste is thermally recovered during disposal. Owing to its high calorific value and low moisture content, the textile cord is used as an alternative fuel in cement plants and cogeneration plants [15].

Steel from tyres constitutes a valuable feed for metallurgical furnaces, where it is reused for steel production. Nevertheless, owing to the well-developed surface and rubber contaminant content, steel scrap from tyre recycling poses a certain risk of strong flames occurring during furnace loading and the high temperature of the furnace gas during charge melting, which might result in filter damage in the flue gas dedusting system. Therefore, recycled tyre steel needs to be of high purity (rubber content <1%) for safe use in steelmaking furnaces [16,17].

As a result of material recycling, a mixture of non-homogenous steel fibres was obtained. After cleaning the fibres of rubber residues and textile cords, they can be used as dispersed reinforcements in concrete. Steel is among the main components of tyres, with contents ranging from 14% to 25% [18].

Most common is the material recycling of tyres, which produces steel fibres of irregular shapes, lengths and diameters. Employing tyre steel fibres as a dispersed reinforcement in concrete seems to be the best way to reuse them. Numerous studies focus on their use as dispersed reinforcement in concrete [17,19,20,21,22,23]. Analysing the impact of RTSFs on the reinforcement efficacy of the brittle cement matrix appears to be the key aspect. Depending on the fibre acquisition method, their geometric parameters, the content of rubber contaminants, and—first and foremost—the content in concrete, the study results vary but are mostly positive. This confirms the feasibility of the fibres being used as dispersed reinforcement. Numerous studies have revealed that concrete containing recycled tyre steel fibres achieves higher flexural strength and other parameters related to fibre-reinforced concrete cracking compared to non-reinforced concrete or those containing commercial steel fibres. The efficacy of concrete reinforcement with recycled tyre fibres increased for higher fibre contents in the concrete and their adequate purity, that is, their lowest possible contamination with rubber [17,24,25,26,27,28,29,30,31,32,33].

It has been proven that a mixture of hybrid steel fibres of different diameters, lengths, and shapes can be more effective than steel fibres of uniform shape and dimensions [2,17,28,34,35,36,37,38,39,40]. The presence of rubber impurities and steel dust in raw recycled tire fibres reduces their adhesion to cement paste. For this reason, and because of the ubiquitous presence of short fibres, they should be added in greater quantities to concrete [17,28,39,40,41]. Some studies have indicated that primary raw steel fibres from used tyres can potentially be used to produce sustainable and durable concrete [42].

Used tyres can be subjected to energy recycling, which means incinerating entire or cut tyres in cement or cogeneration plants as an alternative fuel. Some sources suggest that energy recycling is the best tyre disposal method, as tyre rubber production consumes 3–4 times more energy than that recovered in energy recycling [43]. Consequently, using recycled rubber for primary purposes rather than incinerating is justified from economic and environmental points of view. Some reports indicate that tyre recycling is more than four times more beneficial than incineration [44]. Poland has postulated a ban on incinerating whole tyres in cement plants in favour of incinerating waste from tyre recycling. Additionally, Poland has proposed introducing solutions based on the Scandinavian circular economy model in municipal heat plants, where waste is first subjected to material recovery.

A growing interest in this topic, supported by the positive results of strength tests on concrete containing recycled tyre fibres, has contributed to attempts to produce recycled tyre fibres on an industrial scale. Primarily recycled tyre fibres form a mixture of fibres of various lengths and diameters and are contaminated with rubber residues. The non-uniform geometric parameters of recycled tyre steel fibres, which prevents control of their lengths and diameters, are the main disadvantage of their use as concrete reinforcements. In some countries, tyre fibres are produced through special technology involving obtaining treated uniform fibres with 0.22 mm diameter in three lengths, i.e., 25, 20, and 15 mm [45]. Additional refining of recycled tyre fibres to treat them and make their dimensions uniform offers the possibility of controlling their geometric characteristics, promoting their marketing, and improving their reinforcement efficacy. However, the treatment entails higher energy consumption and greenhouse gas emissions related to their preparation and generates higher treatment-related costs. From an environmental viewpoint, it is crucial to investigate the possibility of using primary tyre fibres on a real scale according to circular economy principles.

In addition to geometric characteristics, dispensing tyre fibres into the concrete mix in a ready-mixed concrete plant is another problem related to their use on an industrial scale. Primarily recycled tyre fibres are a mixture of various lengths, diameters, and non-uniform shapes, which can cause tangling and clustering. The authors’ previous studies [17,19] analysed scientific papers involving only laboratory tests and manual dosing of tyre fibres into the concrete mix, guaranteeing their gradual separation before addition to ensure their uniform distribution. An analysis of the possible dispensing of recycled tyre fibres on a real scale in a ready-mixed concrete plant are presented in this paper, which is a new approach in publications on the subject matter. The authors did not find any literature on industrial-scale tests.

Evaluation of the need to process recycled tyre fibres to ensure effective fibre reinforcement of concrete in line with the principles of the circular economy is the aim of this article. An attempt to implement recycled tyre steel fibres on an industrial scale without further treatment to reduce product emissivity is described in this paper. Replacing industrial steel fibres with recycled tyre fibres in fibre-reinforced concrete has been demonstrated to reduce the index of greenhouse effect potential by ca. 30% [2]. Finding a balance between environmental friendliness and product durability is among the challenges of a circular economy.

All tests presented in this study were performed for concrete produced on an industrial scale. The investigated properties of concrete mixes and concrete, which are measures for evaluating the fitness of fibres for concrete reinforcement, included the concrete mix slump test of its consistency, air content in concrete mixes, density of concrete mixes, compressive strength after 2, 7, and 28 d, flexural strength after 2, 7, and 28 d, flexural tensile strength after 2, 7, and 28 d, modulus of elasticity in compression after 28 d, residual flexural tensile strength, and fibre distribution using computer tomography.

The authors’ studies on the use of recycled tyre fibres as dispersed concrete reinforcement [17,19] are continued in this article, revealing that treated tyre steel fibres can effectively strengthen the brittle cement matrix when they are long enough, dispensed in the right quantity, and uniformly distributed in the cement mix.

## 2. Materials and Methods

### 2.1. Materials

**Figure 1:**
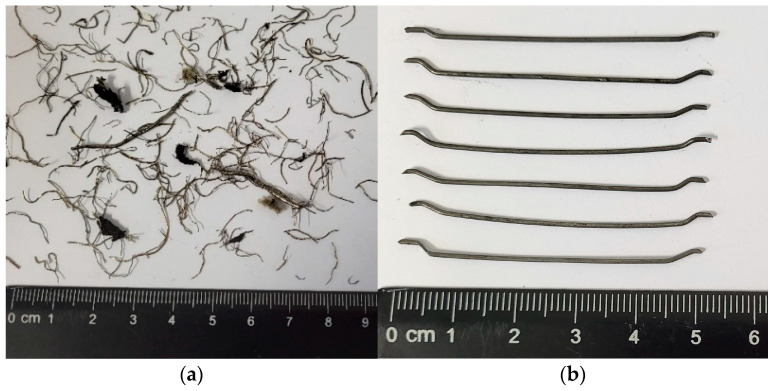
Pictures of steel fibres: (**a**) recycled tyre steel fibres, (**b**) manufactured steel fibres.

Two types of steel fibres were selected for the tests, that is, non-treated recycled tyre fibres, not subjected to additional RTSF treatment, and—for comparison—hooked 50 mm long and 0.8 mm diameter MSFs. Photographs are shown in Figure 1.

Fibre characteristics are summarised in Table 1.

**Figure 2:**
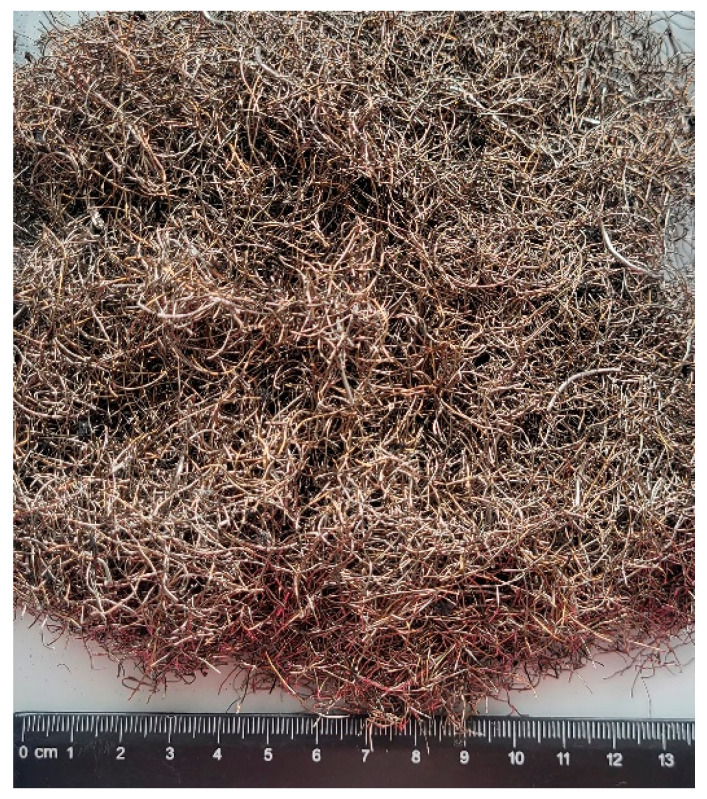
Mixture of raw recycled tyre steel fibres, RTSFs.

MSFs are commonly used as dispersed concrete reinforcements and conform to standard requirements [46]. The standard quantity of MSFs in concrete amounts to 15–45 kg/m^3^. RTSFs originate from the primary mechanical treatment of tyres, with no additional treatment. The contents of fine rubber pieces, textile contaminants, and steel dust were ca. 3%. Contaminated fibres not subjected to extra treatment were selected for the tests to investigate the possibility of their industrial implementation and the lowest possible environmental impact. Additional refining, treatment, and segregation depending on the dimensions will use extra energy, contributing to a higher carbon footprint of fibre production and increased costs. Hence, this study involved attempts to dispense RTSFs on an industrial scale in a ready-mixed concrete plant. The method of dispensing tyre fibres on an industrial scale can prevent their implementation in production. Recycled tyre fibres are a mixture of entangled fibres of various shapes and dimensions, resulting in clustering and dispensing difficulties. An image of the RTSF mixture is shown in Figure 2. Previous tests [17,19] were performed in a laboratory, and the fibres were manually separated, which is impossible in industrial applications. Therefore, dispensing RTSFs may cause technological problems and limit their industrial use.

Comparative tests were performed by adding RTSFs and MSFs to concrete. For industrial-scale tests in ready-mixed concrete plants, a concrete formula was designed with the added RTSFs and MSFs. This was assumed to be flooring concrete, as this is the primary intended use of RTSFs. Table 2 summarises the concrete composition.

Concrete parameters:

– Strength class C25/30;
– Water/cement ratio 0.50;
– Consistency class S3.

**Figure 3:**
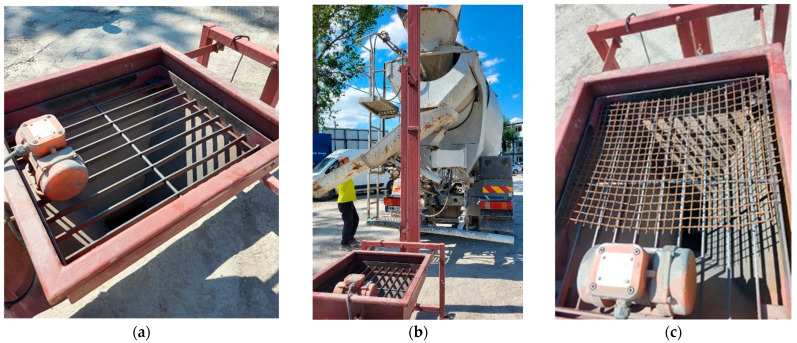
System for steel fibre dispensing into a concrete delivery truck: (**a**) system for manufactured steel fibres; (**b**) dosing fibres to the concrete mixer; (**c**) system for recycled tyre steel fibres.

Concrete production in the plant was as follows: coarse aggregate is mixed with sand. Then, cement, water, and admixtures are added. The whole is mixed for about 1 min. Then, the concrete mixture is poured into a concrete truck, which drives up to the fibre dosing device (Figure 3). Steel fibres are dosed into the concrete truck, then all the ingredients are mixed for about 10 min.

RTSFs were added to the concrete at 25 kg/m^3^ and 45 kg/m^3^, whereas the addition of MSF amounted to 15 kg/m^3^. RTSF quantities were higher by approximately 65% and 200%, resulting from the fact that the fibres were obtained with primary technology, not additionally refined, and severely contaminated with rubber particles, with a significant share of short and ineffective fibres. Moreover, most RTSFs are ca. half as long as, and their diameters half the size of, those of MSFs; a significant number of fibres are short and ineffective in concrete. Therefore, it is necessary to add more untreated tyre fibres. The added quantities of MSFs were minimal (15 kg/m^3^), following the initial type tests to fulfil the strength requirements according to EN 14889-1 [46]. The symbols used are listed in Table 3.

### 2.2. Methods

Concrete mixes were prepared in a ready-mixed concrete plant. This part aimed to develop a method of dispensing recycled tyre fibres into a concrete delivery truck to achieve a uniform distribution of fibres without clusters that reduce reinforcement efficacy.

The following tests were performed after producing the mixture with RTSF and MSF fibres:

- The consistency of fresh concrete by the slump test according to EN 12350-2 [47].
- The test involves placing the mixture in a mould (Abrams cone) and then removing the cone. The difference in the height of the mould and the slump of the mixture is a measure of consistency.
- Air content in compacted fresh concrete according to EN 12350-7 [48].
- The method of testing involves measuring the air content by pressure in a vessel with a volume of 5 L.
- Density of compacted fresh concrete according to EN 12350-6 [49].
- The method of testing consists of measuring the mass of a concrete mix placed in a container of known volume, e.g., 5 L. The same container used for testing the air content is used for the test.
- Compressive strength after 2, 7, and 28 d according to EN 12390-3 [50].
- The test was carried out on three 150 mm cube samples for each test term. Compressive strength is the ratio of the maximum compressive load to the compressed surface area.
- Flexural strength after 2, 7, and 28 d according to EN 12390-5 [51].
- The test was carried out on three cuboid samples with dimensions of 150 × 150 × 700 mm, for each test term. The test was performed in a four-point loading system. The specimens were subjected to a bending moment through the lower and upper rollers.
- Splitting flexural tensile strength after 2, 7, and 28 d according to EN 12390-6 [52].
- The test was carried out on three sample cubes with 150 mm sides. A cubes specimen was subjected to a compressive force applied to a narrow region along its length. The test consists of splitting a cube by a load applied along the generatrix.
- Modulus of elasticity in compression after 28 d according to EN 12390-13 [53].
- The test was performed on six cylindrical samples, 100 mm in diameter and 200 mm in height, drilled from large concrete elements manufactured in a batching plant. For three of the accompanying samples, compressive strength was measured, and for the remaining three samples, the stabilised modulus of elasticity in compression was measured. For each of these samples, three extensometers were mounted to measure displacement.
- Residual flexural tensile strength according to EN 14651 [54].
- The residual flexural tensile strength after 28 days was tested according to EN 14651 [54] as a three-point bending with a notch. The test was performed on eight 150 × 150 × 550 mm specimens for each mix, with about a 4 mm wide and 25 mm deep notch in the middle of the beam for the purpose of testing the propagation of the beam crack’s location. The residual flexural tensile strength test result is the average of the eight specimens. During bending, the load (kN) and CMOD (mm) were read at a frequency of 5 Hz up to CMOD = 3.5 mm, allowing approximately 5200 load/CMOD results to be read. The crack occurred at the notch and propagated through the beam cross-section concerning all specimens tested. The first fracture (proportionality limit) was determined according to EN 14651 [54] as the highest load at the CMOD from 0 to 50 µm.
- Fibre distribution using X-ray computed tomography.
- Core samples (100 mm diameter and height) drilled from larger concrete volumes produced in a batching plant were used to test the fibre distribution in the concrete.
- The test was performed to observe the distribution of recycled tyre steel fibres in hardened concrete and check if the fibres are uniformly distributed in the concrete mix during mixing. The test was conducted using General Electric (GE V|TOME|X M300) computer tomography system, rendering 3D images of the fibre distribution in core samples.

## 3. Results

### 3.1. RTSF Dispensing on a Technical Scale

As shown in Figure 2, primary RTSFs have the form of a compact mixture of thin fibres of various shapes. Their dispensing under laboratory conditions did not pose a major problem, as they could be manually separated before being added to the concrete mix. In the case of industrial implementation, separating RTSFs to ensure their uniform dispensing and creating a homogenous concrete mix can be a challenge. Commercial steel fibres are dispensed into a concrete delivery truck in a ready-mixed concrete plant with a special dispensing system, that is, a shoot machine whose vibrations separate the fibres and dispense them gradually into the concrete mix. The method of dispensing MSFs into a concrete delivery truck using a special system is shown in Figure 3a,b. The common dispensing system might not have been relevant for recycled tyre fibres, some of which are very short, as clusters of RTSFs can penetrate through wide slots and form clusters called “hedgehogs” in the concrete mix. That is why, in order to uniformly dispense recycled tyre fibres, a dense grid was placed on a common shoot machine. Its vibrations broke the RTSF clusters, enabling adequate dispensing of RTSFs to the concrete delivery truck. Figure 3c shows the RTSF dispensing method for a concrete delivery truck.

### 3.2. Results of Concrete Mix Tests

Table 4 summarises the test results for the concrete mix with the MSFs and RTSFs.

The characteristics of the concrete mix with an RTSF content of 25 kg/m^3^ did not deviate from those of the mix with 15 kg/m^3^ MSFs. The consistency, density, and air content were similar. The workability, homogeneity, and viscosity of the concrete mix in the RTSF-25 samples were similar to those of the MSF-15 sample containing traditional steel fibres.

Significant differences in the properties were observed for the RTSF-45 mix. An approximately 70% higher air content was observed compared to MSF and RTSF-25, which caused a density drop and an increase in the RTSF-45 mixture’s consistency compared to other mixes. High air entrainment in the RTSF-45 mixture could be caused by compromised concrete structure by a large amount of raw, uncleaned RTSF fibres, fibre entanglement and the presence of impurities. The primary contaminated RTSFs dispensed at higher quantities deteriorate the characteristics of the concrete mix and jeopardise structural stability and workability. Therefore, their use in concrete should entail stringent control of the concrete quality. An air entrainment value of 7.5% may prevent the achievement of a smooth surface during concrete flooring smoothing. Typically, as much air as possible should be evacuated from flooring concrete, and high aeration of the RTSF-45 mix is not desired. Moreover, it deteriorates the strength characteristics of concrete.

### 3.3. Results of Hardened Concrete

**Figure 4:**
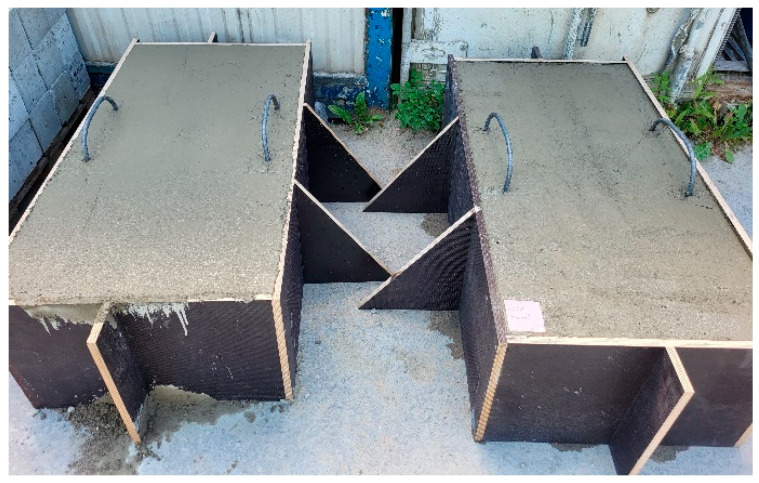
Large concrete moulds sized 160 × 40 × 60 cm.

The manufactured concrete mixes containing RTSFs and MSFs were used to produce concrete samples for strength tests and poured into dedicated larger moulds (160 × 40 × 60 cm) to observe the RTSF and MSF distributions in a larger volume of manufactured concrete. The large moulds are shown in Figure 4.

The RTSF-25 sample properties suggest the possibility of feeding concrete with a pump; the mixture was easily smoothed. The resulting surface was smooth, with no air pores and no difference from the surface obtained for the MSF-15 reference concrete mix. The properties of the MSF-15 and RTSF-25 mix for the concrete mix quality, concrete mechanical characteristics, and the possibility of embedding concrete in the structure can be considered identical.

**Figure 5:**
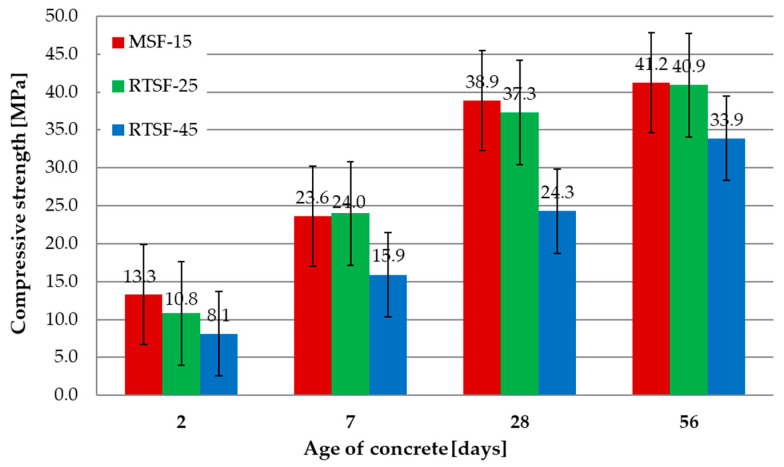
Results of compressive strength tests.

Figure 5 shows the results of the compressive strength tests after 2, 7, 28, and 56 d.

The concrete containing 25 kg/m^3^ of non-treated RTSFs achieved compressive strength test results similar to those of concrete containing 15 kg/m^3^ of MSFs, revealing the potential for using non-treated tyre fibres. The amount of RTSFs (10 kg/m^3^ more) was caused by the presence of contaminants and fibres shorter than the MSFs. The addition of 45 kg/m^3^ of RTSFs resulted in reducing the compressive strength after 2, 7, 28, and 56 d compared to both RTSF-25 and MSF-15. The decrease was significant enough (ca. 35% after 28 d and ca. 17% after 56 d) that the requirements for the C25/30 compressive strength were not fulfilled. The decrease is caused by the compromised concrete structure and significant aeration of the concrete mix by too many fibres.

The mean compressive strength results for MSF-15 and RTSF-25 are comparable; after 2, 7, 28, and 56 d of curing, the results were similar, revealing the potential for using non-treated tyre fibres. Increasing the amount of tyre fibres to 45 kg/m^3^ deteriorated the compressive strength by ca. 25–35% compared to RTSF-25 concrete.

**Figure 6:**
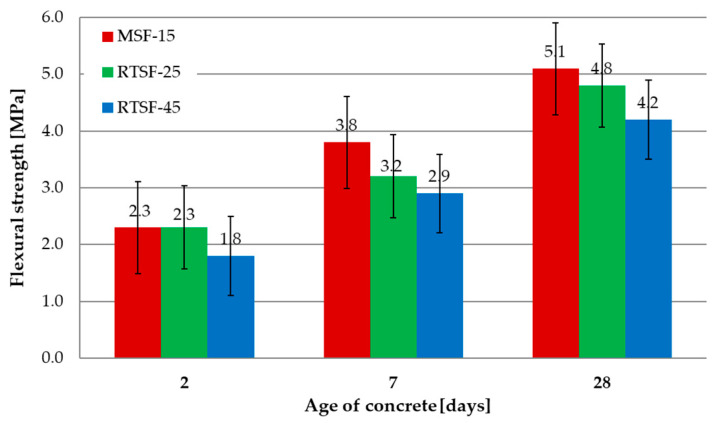
Results of flexural strength tests.

Figure 6 shows the results of the compressive strength tests after 2, 7, and 28 d.

The flexural strengths of MSF-15 and RTSF-25 concrete after 2 d were equivalent and slightly lower than that of RTSF-45 concrete. After 7 and 28 d, the flexural strength of concrete containing RTSF fibres was lower than that of concrete containing MSF-15 fibres. It can be concluded that the flexural strength results for RTSF-25 and MSF-15 were high for the designed C25/30 class of concrete based on gravel aggregates. Although the quantities of contaminated RTSFs are more significant than those of MSFs, the RTSFs do not increase the flexural strength owing to the presence of contaminants and short fibre lengths, as well as air entrainment in the mix caused by the fibres. For RTSF-45 concrete, they were ca. 10–20% lower than those of RTSF-25. This can be caused by compromised structure resulting from concrete mix aeration by raw tyre fibres.

**Figure 7:**
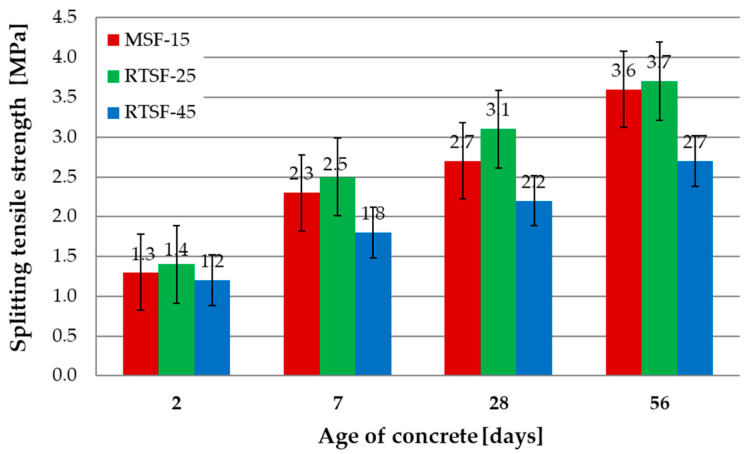
Results of splitting flexural strength tests.

Figure 7 shows the results of the splitting tensile strength tests after 2, 7, 28, and 56 d.

The tensile strength tests at splitting revealed slightly higher values for RTSF-25 concrete than for MSF-15 concrete. Similar to previous tests, an increased amounts of raw tyre fibres resulted in deteriorated concrete characteristics. Similar to previous tests, increasing the amount of raw tyre fibres to 45 kg/m^3^ resulted in deteriorated characteristics of the hardened concrete. The test of the tensile strength at splitting after 56 d for RTSF-25 and MSF-15 conforms to the requirements for airport surfaces, confirming its application potential.

The test results for early compressive strength and early test results for flexural strength and tensile strength at splitting provide vital information on the concrete’s similar tensile strength. This is significant for concrete structures or fibre-reinforced concrete, as it confirms the similarity of MSF-15 and RTSF-25 concrete’s resistance to shrink cracking.

**Figure 8:**
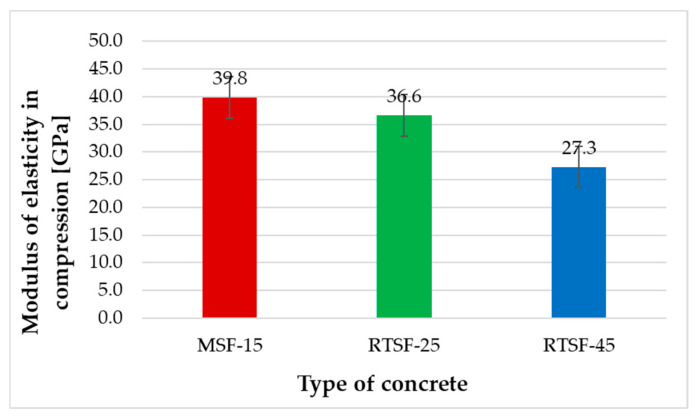
Results of modulus of elasticity in compression.

Figure 8 shows the test results for the modulus of elasticity after 28 d.

The modulus of elasticity of the samples manufactured in a batching plant revealed that MSF-15 concrete containing 15 kg/m^3^ of commercial hooked fibres achieved the highest results. The results for RTSF-25 concrete were 8% lower, and those for RTSF-45 concrete were 31% lower. A slightly higher result for Young’s modulus test for concrete containing RTSF-25 compared to MSF-15 can result from contaminant content but can become an advantage for flooring concrete. Concrete with a lower Young’s modulus exhibited lower shrinkage potential. This is a favourable feature for concrete and fibre-reinforced concrete structures as it improves energy absorption and helps sustain dynamic loads.

**Figure 9:**
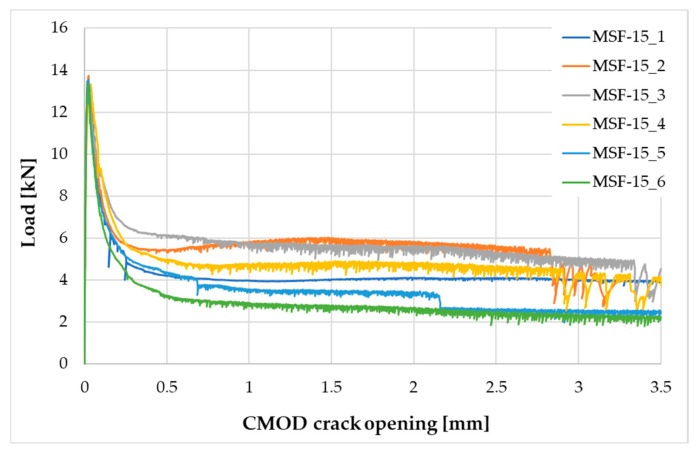
Load–CMOD relationship for the MSF-15 concrete.

**Figure 10:**
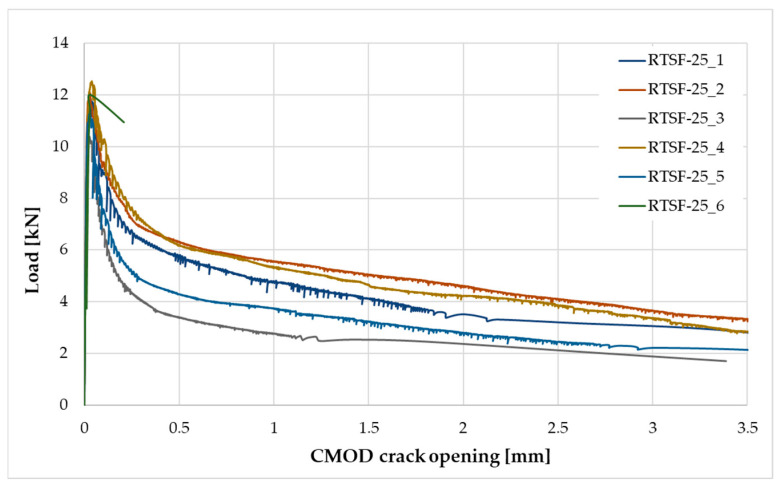
Load–CMOD relationship for the RTSF-25 concrete.

**Figure 11:**
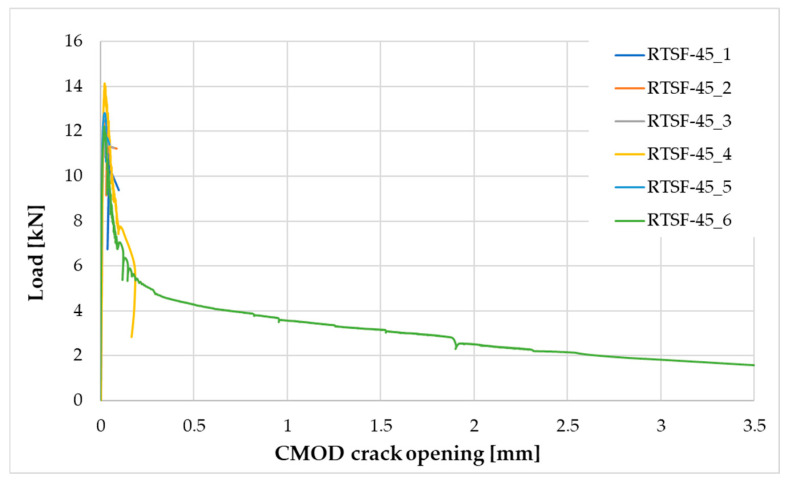
Load–CMOD relationship for the RTSF-45 concrete.

Residual strength results are shown in Figure 9, Figure 10 and Figure 11 and are summarised in Table 5.

The residual strength test results for MSF-15 and RTSF-25 can be considered similar; the test is highly sensitive to performance errors, and its results strongly depend on the fibre distribution in the concrete beam. The RTSF-25 concrete reached slightly lower residual flexural tensile strength values, but the fibres transferred the loads, and only one out of the six beams cracked. The result of the flexural tensile strength at the LOP being lower for the RTSF-25 sample than for MSF-15 can be attributed to contaminated recycled tyre fibres. The RTSF-25 concrete reached a higher residual flexural tensile strength value for CMOD = 0.5 mm and slightly lower values for CMOD = 1.5, 2.5, and 3.5 mm than the MSF-15 concrete, confirming the effective reinforcement of the brittle cement matrix by the treated recycled tyre fibres.

The residual flexural tensile strength test results revealed that non-treated RTSFs with an amount of 45 kg/m^3^ did not transfer the load along the cement matrix crack. Brittle fractures occurred in five of the six tested samples. Large amounts of contaminated tyre fibres deteriorate the strength characteristics of concrete and are inefficient when added in high quantities and are ineffective as dispersed reinforcement.

**Figure 12:**
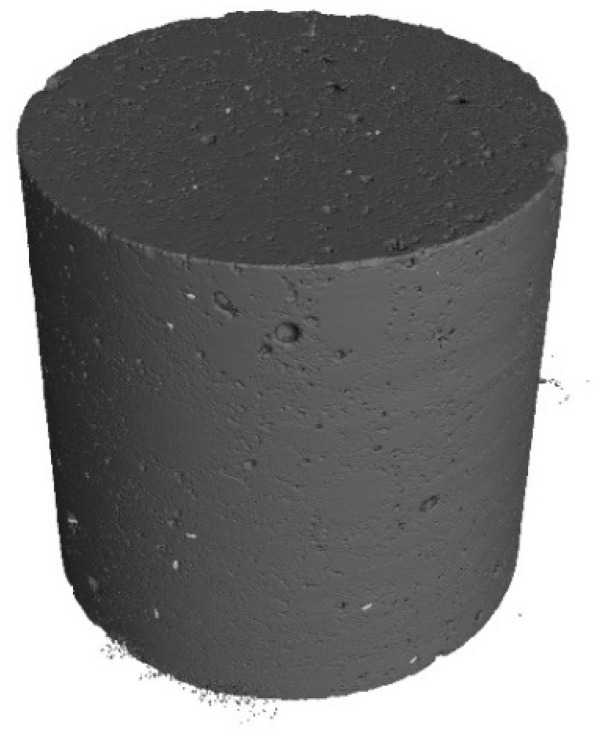
MSF-15 test sample.

**Figure 13:**
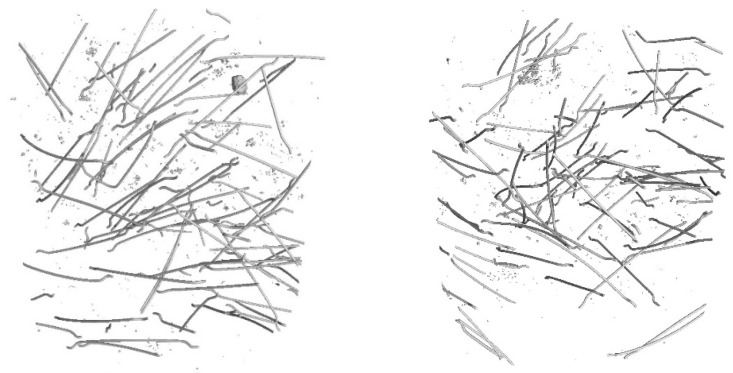
CT-generated image—distribution of MSF-15 fibres.

**Figure 14:**
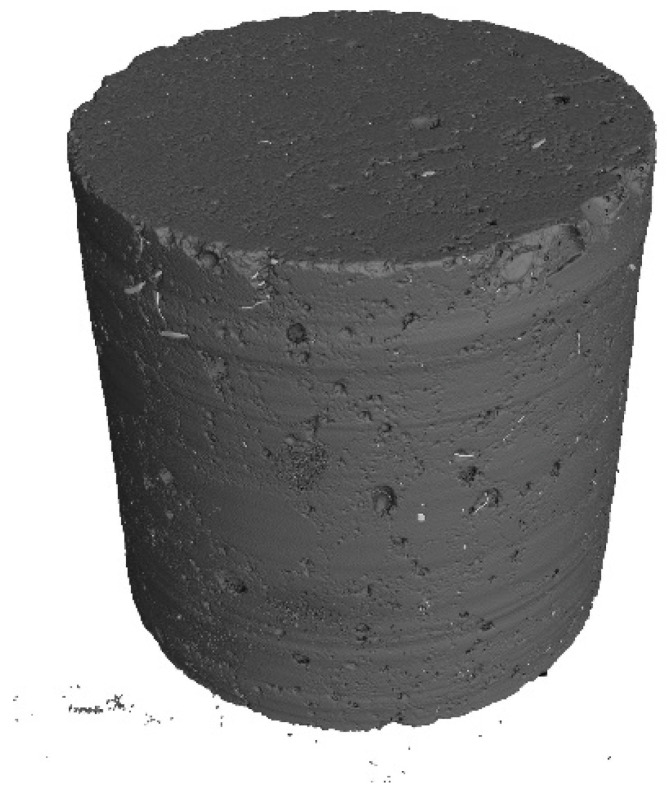
RTSF-25 test sample.

**Figure 15:**
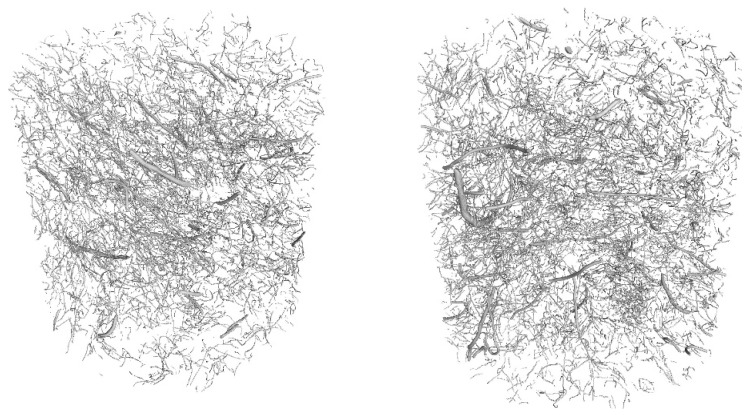
CT-generated image—distribution of RTSF-25 fibres.

**Figure 16:**
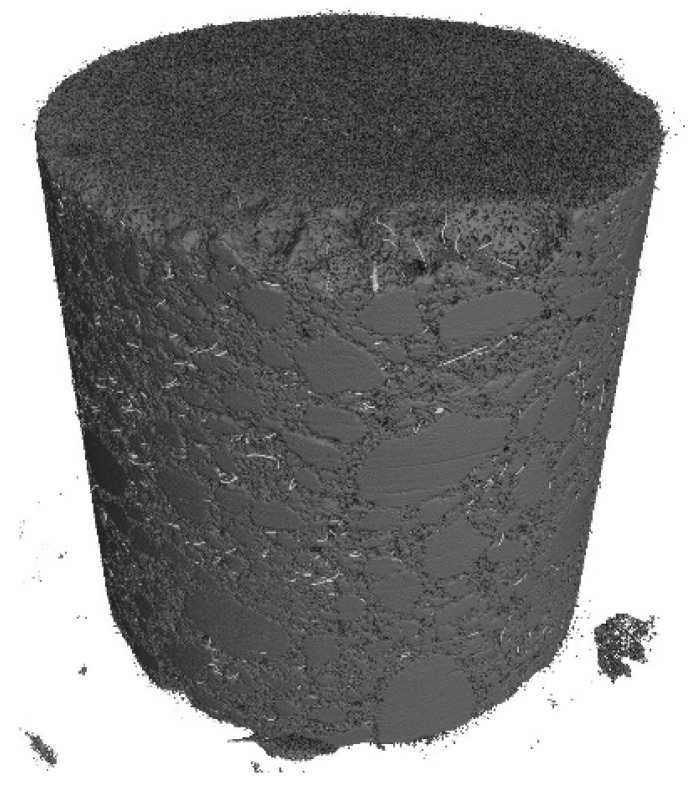
RTSF-45 test sample.

**Figure 17:**
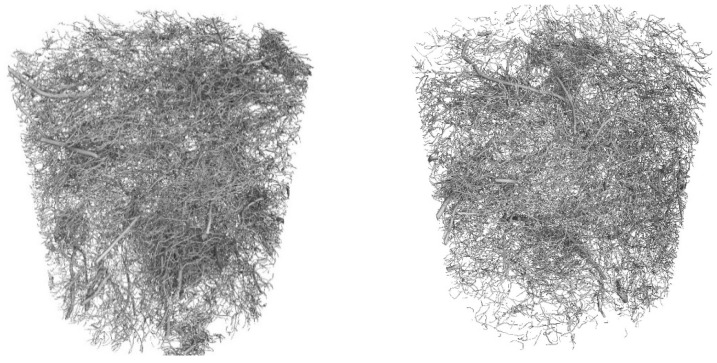
CT-generated image—distribution of RTSF-45 fibres.

Core samples (100 mm diameter and height) drilled from larger concrete volumes produced in a batching plant were used to test the fibre distribution in the concrete. They are shown in Figure 4. Performing the test on pre-formed samples might not illustrate the exact fibre distribution in concrete owing to the mould sizes. Collecting core samples from larger concrete volumes presents the actual fibre distribution in the concrete volume. The tests were performed using computed tomography. Figure 12, Figure 13, Figure 14, Figure 15, Figure 16 and Figure 17 show the test results for the fibre distribution in the concrete.

**Figure 18:**
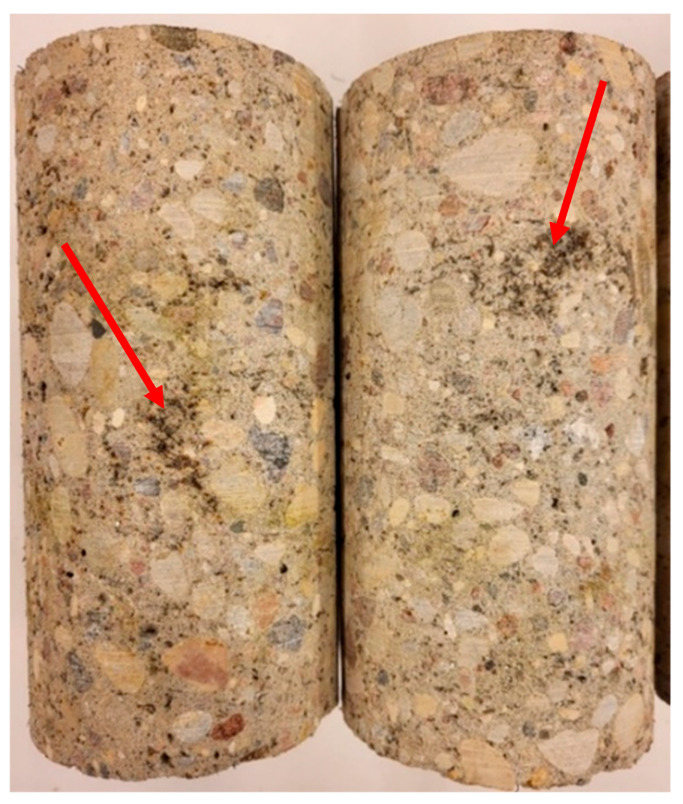
Samples collected from the RTSF-45. The arrows indicate the concrete sample defects caused by the recycled tyre fibre clusters.

The tests demonstrated that adding excessively high quantities of non-treated fibres, most of which were short and contained rubber and steel dust, resulted in air entraining in the concrete mix and compromising the concrete structure. After cutting out a sample from a larger volume of manufactured concrete, the material containing 45 kg/m^3^ of non-treated RTSFs revealed the presence of large pores and RTSF clusters, as shown in Figure 18. Excessive aeration and compromised structures contribute to reduced concrete strength. The content of rubber and other contaminants and fibres that are too short deteriorates the properties of the concrete mix and cured concrete. The fibres in the samples cut out from larger volumes and marked as MSF-15 and RTSF-25 were well distributed, and no fibre clusters were reported. Large air pores can be observed on the RTSF-45 sample cut from a larger volume of manufactured concrete, and the fibres were non-uniformly distributed. Using 45 kg/m^3^ of recycled tyre fibres, owing to the high aeration and the presence of local clusters (agglomerates called “hedgehogs”) of numerous fibres, as well as the content of rubber and other contaminants, deteriorates the concrete strength characteristics and negatively impacts the possibility of embedding the mix correctly in floor structures.

Based on the results, it was concluded that using large quantities of non-treated recycled tyre steel fibres entails the risk of adverse impacts on the concrete mix properties due to air entrainment. The same applies to hardened concrete, the structure of which becomes inhomogeneous, and the strength parameters of which deteriorate.

The strength properties of concrete reinforced with steel fibres are influenced by their geometrical properties (length, diameter, profile) and adhesion to cement paste. Previous studies by the authors show that cleaned and processed fibres from recycled tires have very good adhesion to cement paste [17,19]. In this article, raw fibres with the presence of impurities and non-uniform in terms of length, also containing short fibres, were studied. The mechanism of action of raw fibres is different from that of cleaned fibres. More raw fibres should be added than industrial fibres, due to the presence of short fibres and impurities. The amount of raw fibres from recycled tires must be strictly controlled, because too large an amount of fibres can disrupt the structure of the concrete mix, creating fibre clusters or air entrainment, which affects the deterioration of strength properties. This mechanism was observed for RTSF-45 concrete, where the compressive strength, bending strength, splitting strength, modulus of elasticity, and residual strength were reduced compared to RTSF-25 and MSF-15 concretes. Short fibres from recycled tires are not effective reinforcement and do not transfer loads, and contamination worsens the adhesion of fibres to the cement matrix.

## 4. Conclusions

According to the circular economy concept, the reuse of waste or recycled materials is a major challenge for the industrial sector. On one hand, industry should use as much waste material as possible. On the other hand, the performance properties and safety of constructions based on waste materials are also important.

The possibility of using non-treated recycled tyre fibres as a dispersed concrete reinforcement is presented in this paper. The novelty in this article is the production of concrete with recycled tire fibres on a technical scale in a ready-mixed concrete plant and the presentation of a method of dosing tire fibres to a concrete mixer. Industrial-scale tests confirmed the possibility of using recycled tyre fibres as reinforcement for concrete structures. The author’s previous studies and other analysed papers were based on laboratory-scale tests, where tyre fibres were manually dispensed into a laboratory mixer. The presented method of dispensing the right quantities of tyre fibres to a concrete delivery truck ensures their uniform distribution and highlights their application potential. The literature does not mention full-technical scale tests for flooring concrete using recycled tyre fibres.

The paper presents the results of tests on concretes with the addition of raw recycled tyre steel fibres dosed in two quantities to concrete (25 kg/m^3^ and 45 kg/m^3^). As a comparison, concrete was made with the addition of 15 kg/m^3^ of manufactured steel fibres. The properties of the concrete mix (consistency, density, air content) and the characteristics of hardened concrete (compressive strength, flexural strength, splitting strength, modulus of elasticity, residual flexural tensile strength, fibre distribution in concrete) were tested. The results indicate that concretes with 25 kg/m^3^ of raw recycled tyre steel fibres exhibit similar strength properties to the reference concrete. However, with the addition of 45 kg/m^3^ of raw tire fibres, the strength properties deteriorated due to the aeration of the mix by the fibres and the disruption of the homogeneous structure.

This study demonstrates that raw, non-treated tyre fibres can be used as dispersed concrete reinforcements. However, their quantity in concrete and its impact on the concrete mix and hardened concrete should be monitored, as the reinforcing efficacy does not always increase with higher quantities of raw recycled tyre fibres. The results obtained for the MSF-15 and RTSF-25 concrete revealed similar characteristics and the possibility of using non-treated recycled tyre fibres. Using almost twice as much raw recycled tire fibres is still preferable to using industrial steel fibres with a high carbon footprint. At higher quantities of raw recycled tyre fibres, as in the RTSF-45 sample, a high aeration was achieved along with a decrease in the concrete strength characteristics. The quantity of recycled tyre fibres should be selected experimentally depending on the fibre length and contamination content. Moreover, industrial-scale tests confirmed the possibility of obtaining good performance (identical to that of the reference flooring concrete), achieving a smooth surface and similar mix characteristics, feeding with a pump, and obtaining uniform fibre distribution in concrete.

The decision to use treated or non-treated tyre steel fibres should be based on the intended use of the concrete. It is always preceded by initial concrete tests and improved checks for reinforced concrete production. Both solutions help to save natural resources during high-emission steel production and match the concept of a circular economy. Finding a balance between environmental aspects and construction durability is key. Using fibre-reinforced concrete with recycled tyre fibres is also vital. For applications where load-bearing capacity and high reinforcement efficiency are maintained, and considering the structural safety, additional treatment, and ensuring greater uniformity of tyre fibre production, using the full potential of fibres in cement composites seems valid. Adequate amounts of raw primary fibres can be used for standard concrete that is not exposed to severe loads. Concrete mix containing raw recycled tyres steel fibres might find a valuable application in various sectors of the building industry. Concrete floorings might be made using those fibres; however, special attention must be paid to possible impurities of fibres which might come up on the surface causing its contamination, although if it is not a final surface it should not be a problem. Another area of application is the production of precast concrete elements where the flexural strength of an element is crucial, such as, e.g., concrete beams used as lintels. Generally, the use of such concrete with RTSF fibres is seen in structures where increasing tensile strength is important. Heavy, massive structures are another area of application in order to increase the density of concrete.

In summary, raw, primary recycled tyre fibres can be employed as concrete reinforcement to strictly control the concrete mix and cured concrete parameters. They can be used in standard flooring concrete rather than in applications in which the load-bearing capacity and safety of use are crucial.

## Figures and Tables

**Table 1:** Geometric characteristics of the fibres.

Type of Fibre	Length [mm]		Diameter [mm]		Description
MSF	50.0		0.80		Circular cross-section, homogenous hooked steel fibres
RTSF	Interval	Median	Interval	Median	Mixture of fibres of various lengths, diameters and shapes, contaminated with fine rubber particles and textiles (ca. 3% contaminant content)
	2.6–52.3	23.5	0.16–2.19	0.25	

**Table 2:** Concrete composition.

Component	Content (kg/m^3^)
Portland cement CEM I 42.5 R NA	155
Blast furnace cement CEM III A 42.5N LH HSR NA	155
Tap water	155
Natural aggregate 0/2 mm (sand)	730
Natural aggregate 2/16 mm (gravel)	1100
Fluidising admixture (superplasticiser)	2.8
Fluidising admixture (superplasticiser)	0.6

**Table 3:** Tested concretes.

Symbol of Concrete with Fibres	Fibre Content (kg/m^3^)	Fibre Type
MSF-15	15	MSF
RTSF-25	25	RTSF
RTSF-45	45	RTSF

**Table 4:** Results of concrete mix tests.

Concrete Symbol	Consistency with a Slump Test (mm)	Concrete Mix Density (kg/m^3^)	Air Content (%)
MSF-15	120 ± 10	2320 ± 20	4.3 ± 0.4
RTSF-25	110 ± 10	2330 ± 20	4.4 ± 0.4
RTSF-45	160 ± 10	2250 ± 20	7.5 ± 0.4

**Table 5:** Test results for the residual flexural tensile strength.

Concrete Symbol	Flexural Tensile Strength [MPa]				
	at the LOP	Residual Flexural Tensile Strength			
		CMOD_1_ = 0.5 mm	CMOD_2_ = 1.5 mm	CMOD_3_ = 2.5 mm	CMOD_4_ = 3.5 mm
MSF-15	4.22	1.50	1.38	1.28	1.05
RTSF-25	3.74	1.66	1.26	1.01	0.89
RTSF-45	4.08	1.38 *	1.02 *	0.69 *	0.49 *
Measurement uncertainty	±0.34	±0.18	±0.15	±0.16	±0.17

* test results for one sample as brittle fracture of the beam occurred in five samples, and the fibres did not transfer the load.

## Data Availability

The original contributions presented in the study are included in the article, further inquiries can be directed to the corresponding author.
